# Neuroimmune-Driven Neuropathic Pain Establishment: A Focus on Gender Differences

**DOI:** 10.3390/ijms19010281

**Published:** 2018-01-17

**Authors:** Vincenzo Coraggio, Francesca Guida, Serena Boccella, Mariantonietta Scafuro, Salvatore Paino, Domenico Romano, Sabatino Maione, Livio Luongo

**Affiliations:** 1 Department of Experimental Medicine, Division of Pharmacology, University of Campania “L. Vanvitelli”, Naples 80138, Italy; coraggio_vincenzo@libero.it (V.C.); franc.guida@gmail.com (F.G.); boccellaserena@gmail.com (S.B.); salvatore.paino@unicampania.it (S.P.); 2 Department of Anesthesiology, Surgery and Emergency, University of Campania “L. Vanvitelli”, Naples 80138, Italy; mariantonietta.scafuro@unicampania.it; 3 Azienda Ospedaliera di Rilievo Nazionale, “A. Cardarelli”, Via A. Cardarelli, Naples 80131, Italy; domenicoromanorl@gmail.com

**Keywords:** microglia, neuropathic pain, T-cell, allodynia

## Abstract

The role of neuroinflammatory cells in the establishment of neuropathic pain has been investigated in depth in the last few years. In particular, microglia have been shown to be key players in the induction of tactile allodynia, as they release proinflammatory molecules that, in turn, sensitize nociceptive neurons within the spinal cord. However, the role of peripheral immune cells such as macrophages, infiltrating monocytes, mast cells, and T-cells has been highlighted in the last few studies, even though the data are still conflicting and need to be clarified. Intriguingly, the central (microglia) and peripheral (T-cell)-adaptive immune cells that orchestrate maladaptive process-driven neuropathic pain seem to be involved in a gender-dependent manner. In this review, we highlight the role of the microglia and peripheral immune cells in chronic degenerative disease associated with neuro-immune-inflammatory processes.

## 1. Introduction

About 7–8% of the European population is affected by neuropathic pain. This pain is severe in perhaps more than half of that population [1,2]. In humans, neuropathic pain is referred as an abnormal pain sensation that results from a lesion or disease affecting the somatosensory system; different animal models have been reproduced in preclinical studies [3,4,5,6,7]. Neuropathic pain is characterized by the appearance of spontaneous pain with manifestation of abnormal sensory symptoms [8,9,10]. Neuropathic pain can result from a stimulus (hyperalgesia or allodynia), or be independent of the stimulus; the pain is spontaneous and described as burning, firing, or throbbing [7,8,11]. The discharge of pain is triggered by the release of algogenic agents at the site of damage, as well as by the recruitment of primary afferent fibers releasing neuropeptides, including substance P. These neurochemical events form the basis of hyperalgesia, which can originate within the injured area (primary hyperalgesia) and at the surrounding tissues (secondary hyperalgesia). The protraction of the inflammatory process results in central sensitization, which plays a crucial role in pain chronification. Pain perception in response to a thermal or mechanical nonalgogenic stimulus is referred to as allodynia, and may represent the main symptom of neuropathic pain states [12].

Pain is a clinical description (in fact it is not a diagnosis) that requires a demonstrable lesion or a disease that complies with established neurological criteria. Moreover, the term “lesion” is usually used when diagnostic investigations (e.g., imaging, neurophysiological studies, biopsies, laboratory tests) reveal a change from the physiological condition or when there is noticeable trauma. On the other hand, the term “disease” is commonly used when the origin of the lesion is known (i.e., ictus, vasculitis, diabetes mellitus, genetic abnormality). The term “somatosensory” refers to all the information the body itself (including the visceral organs) perceives, while information about the outside world is excluded (e.g., vision, hearing, or smell). Hence, the presence of symptoms or signs (e.g., pain on touch) alone cannot justify a diagnosis of neuropathy (International Association for the Study of Pain (IASP) taxonomy). Common causes of neuropathic pain include pathological processes such as tumor infiltration, neurotoxicity, inflammation/infection, and metabolic abnormalities, as well as traumas such as external lesions or nervous compression. In addition, it may also be due to therapeutic interventions such as surgical operations, chemotherapy, and radiotherapy. Genetic predisposition (hereditary, metabolic/endocrine neurodegenerative alterations) may also play a role [13]. Taken together, neuropathic pain is a debilitating and chronic disease that requires further investigation to understand its molecular and cellular mechanisms, and the development new effective treatments.

Recently, several pathways have been discovered highlighting the important role of neuroinflammation in the development and maintenance of neuropathic pain.

This review highlights the role of central and peripheral immune cell involvement in the neuroinflammatory processes associated with neuropathic pain states, with a specific reference to gender differences.

## 2. Role of the Neuroinflammation in Neuropathic Pain Establishment

A state of neuropathic pain is characterized by the appearance of spontaneous pain with the manifestation of abnormal sensory symptoms, including hyperalgesia and allodynia to thermal or mechanical stimuli. The prolonged/abnormal transmission of pain from the periphery to the spinal cord leads to central sensitization characterized by increased excitability of pain-processing neurons [14,15] and activation of microglial cells and astrocytes [4,16]. These biochemical and cellular responses of the nervous system to damage are commonly indicated as neuroinflammation.

Increasing evidence suggests that neuroinflammation is an underlying cause of several central nervous system (CNS) diseases including neuropathic pain but also Alzheimer’s disease, Parkinson’s disease, multiple sclerosis, and psychiatric disorders [17]. It is clear that there are differences between CNS neuroinflammation occurring in neurodegenerative diseases (e.g., multiple sclerosis) and what is manifested in chronic pain. Neuroinflammation in neurodegenerative diseases is in most cases the result of direct CNS damage, which causes further neuronal degeneration and cell death (secondary lesions) [17]. In neuropathic pain, however, neuroinflammation is caused by peripheral damage and by the excessive neuronal activity of primary sensory neurons. Hence, CNS neuroinflammation after peripheral injury appears to be relatively mild and does not cause huge neuronal loss as compared to that presented in neurodegenerative diseases [18,19,20]. However, differences in the role of proinflammatory cytokines in neurodegenerative disease compared to inflammatory pain should be emphasized. Cytokines, such as tumor necrosis factor alpha (TNF-α) and interleukin 1 beta (IL-1β), cause neurodegeneration and compromise both memory and synaptic plasticity in different regions of the brain (e.g., the hippocampus and toothed spine) that are associated with brain impairment in neurodegenerative disease [5,21,22]. On the other hand, TNF-α and IL-1β act as neuromodulators following peripheral nerve injury and in this case induce/increase synaptic plasticity as well as inflammatory and neuropathic pain. Indeed, increased IL-1β/TNF-α released by microglial cells and astrocytes at the spinal cord level, as well as in the brain, was observed in neuropathic mice [23,24]. In fact, in both human and animal studies, a cytokines-mediated immune system imbalance is associated with neuropathic pain development [25,26]. In particular, TNF-α and IL-1 β are considered to be the key chronic pain mediators and thus may represent favorable targets for novel pain treatment strategies [24,27,28,29,30,31].

Interestingly, a promising role for oligodendrocytes in the induction of neuropathic pain has been recently suggested. Indeed, over-expression of the oligodendrocyte-derived IL-33 at spinal levels is linked with the release of other cytokines, including TNF-α and IL-1β, as recently reported by Zarpelo et al., 2016 [32].

## 3. Microglia Role in the Establishment of Tactile Allodynia

The focal point for any discussion of neuroinflammation is microglia. This is because these cells perform primary immune supervision and macrophage-like activities (including the production of chemokines and cytokines) in the CNS [33]. In fact, much of CNS’s innate ability to defend itself is mediated by the microglia. Overall, the microglial cells constitute 10% of the CNS population in the non-neuronal subdomain. Microglial cells have an active role in immune surveillance, including propagation of inflammatory mediators that are transmitted on the periphery [34,35]. These responses have a central role in the coordination between the immune system and brain. For example, in infection or disease, microglia switch to the “activated” phenotype and act as inflammatory cells. Activated microglial cells rapidly change their transcriptional profile and determine the production of cytokines and inflammatory chemokines [34,35]. Depending on the context, their production may facilitate recruitment of leukocytes in the brain [36]. In their activated form, microglial cells undergo cytoskeletal rearrangements that alter the expression pattern of the receptors on the cell surface. Thanks to these alterations, these cells are able to migrate to sites of lesions or infections [37] and potentially increase their phagocyte efficiency [15,38]. In general, activation of microglia and increased expression of cytokines are planned so that they can defend the CNS [34,35]. However, amplified, exaggerated or chronic microglia activation results in pathological changes, but also neuro-behavioral complications that can lead to depression and cognitive deficits [18,39].

The role of microglia in the establishment of tactile allodynia has been largely investigated in several neuropathic pain animal models in the last few decades. It has been shown that microglia change their phenotype after peripheral injury and become activated. Activated microglia, like macrophages in the periphery, can contribute to both allodynia and hyperalgesia by releasing pro-inflammatory molecules in the spinal cord [40,41,42]. After peripheral nerve injury, microglia activation in the spinal cord progresses through a hypertrophic morphology, proliferation, and a change in gene expression. It has been suggested that in both males and females there is a proliferation of microglia after spared nerve injury model of neuropathic pain. However, while there is an up-regulation of the purinoceptor 2X4 (P2X4) ATP receptor in males, this is not evident in females. This different regulation seems to be a key point for the microglia-driven tactile allodynia induction [43,44].

## 4. Role of Peripheral Immune System in the Development of Neuropathic Pain

In recent years, increasing evidence has indicated a pivotal role of the peripheral immune system, in parallel with microglia, in the induction and maintenance of neuropathic pain [25,45,46]. Cytokines and neutrophils have been found to play an important part during the early stages of acute pain, whereas T lymphocytes appear to play a central role in chronic neuropathic pain [47,48]. For example, there is rapid infiltration and transient presence of neutrophils after the lesion, disappearing about 3 days after spinal cord injury [49]. Monocytes are also hooked to the injured spinal cord, with lymphocytes found within the spinal cord after a long period of time, reaching their maximum concentration in the mice after 42 days [50,51] and in humans after several months post trauma [49].Therefore, peripheral immune cells significantly contribute to the inflammatory environment within the spinal cord after injury [39]. Based on available evidence, we can therefore assume that the main elements involved in neuroinflammation are microglia and the infiltrating immune cells.

## 5. Gender-Dependent Immune-Driven Neuropathic Pain

**Figure 1:**
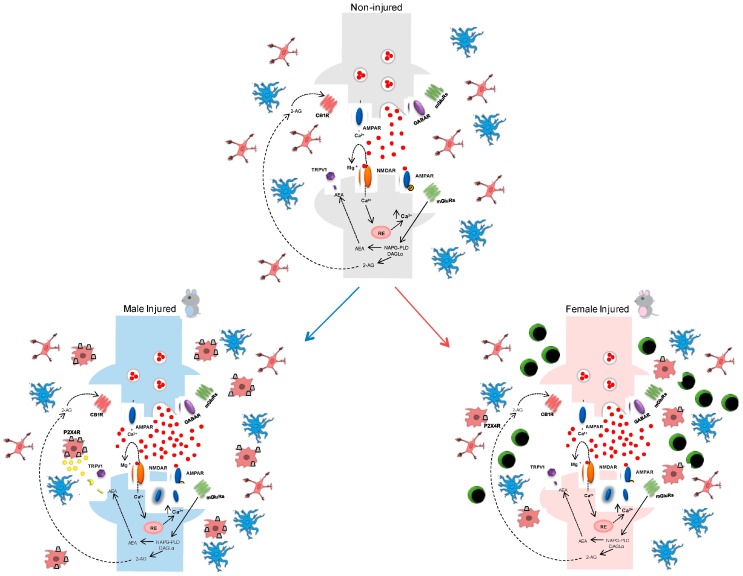
Schematic representation of immune system-related sex differences in injured spinal synapses. The upper panel shows resting microglia (red) and astroglial (blue) cells surrounding glutammatergic synapses under physiological conditions (non-injured). Following nerve injury (neuropathic pain i.e.,), allodynia and neuronal excitability appear at the dorsal horn of spinal cord. These events are mediated by abnormal glutamate, substance P, and ATP release in the synaptic cleft accompanied by microglia activation (red body) with consequent upregulation of P2X_4_R in males (lower-left panel) but not in females. Indeed, in female subjects (lower-right panel), allodynia is mainly associated with T-cell infiltration (green body) at the spinal cord level.

Several recent studies advance the hypothesis that there is a difference between the sexes in the initiation and maintenance of neuroinflammation. In particular, there appears to be a greater involvement of microglia in males, whereas in females neuroinflammation appears to be primarily driven by immune cells [16,51,52]. Women, as compared to men, are more predisposed to developing neurodegenerative diseases [14,53,54,55], migraines, back pain, and osteoarthritis, as well as painful autoimmune disorders like rheumatoid arthritis [52]. In recent years, several studies have focused on sexual dimorphisms that are needed in microglia in both conditions of health and disease. There does not seem to be difference in the two genera at the level of the CNS, while there do seem to be differences at the peripheral level, especially in the proliferation of immune cells [52]. Differences between the sexes with respect to the number, morphology, and molecular phenotype of microglia that occur during development have been reported. These changes could explain, at least in part, the different predisposition that females and males show for some brain pathologies at different stages of development [16,56,57,58,59,60]. However, the mechanisms that underlie the gender differences have not yet been well clarified, particularly with respect to their dependence on genetic or hormonal causes, even if there seem to be no gender differences at the transcriptional level in genes related to nociception [52]. Many studies have focused on the sex hormones estradiol, progesterone, and testosterone, which are known for their ability to reduce inflammation in the nervous system, with multiple and complex mechanisms that partially involve modulation of microglia response to lesions [41,61,62,63,64,65,66]. In the initial stages, the microglial cells migrate into the damaged tissue from other areas, and within the lesion they proliferate and begin to produce a series of molecules for signal transmission. This mechanism determines the recruitment of peripheral immune cells and astrocytes, for example that which occurs in the activation of astrocytes and in the formation of scars [67,68,69] (Figure 1).

One of the models used to study the neuroinflammatory processes is of cortical wound injury. In fact, this model is used for the analysis of multicellular and spatiotemporally orchestrated reactions occurring in neuroinflammation [70]. Also, gender differences have been reported in this model. Indeed, in males there was a greater density of ionized calcium-binding adapter molecule 1 (Iba1) immunoreactive cells in the edges of the lesion than females, suggesting a greater proliferation or migration of microglia cells towards the lesion site in males in response to the same type of injury. It should be noted, however, that differences observed between the sexes regarding the quantity of immunoreactive Iba1 cells at the bounded site may be due to a different peripheral immune cell infiltration rate, since Iba1 is expressed not only by microglia, but also some populations of macrophages. Given that there is a breakdown of the blood–brain barrier (BBB) in cutting wounds, a large invasion of peripheral blood cells in the cerebral parenchyma could be facilitated [61].

In addition to the density of Iba1 immunoreactive cells in the cut wound model, the expression of the neuroprotective protein neuroglobin has also been studied. Neuroglobin has been described as an endogenous neuroprotective molecule in response to hypoxic/ischemic insults [6,44,46,64,71], but it can also occur after traumatic brain injury [32,72,73] or a spinal cord lesion [74]. It has been correlated to a higher neuronal survival and also to a functional improvement, since the neuroglobin has antioxidant as well as anti-apoptotic and anti-inflammatory properties [75,76,77,78]. The regulation of neuroglobin expression has been identified as one of the molecular mechanisms that mediates estradiol in neuroinflammation, in particular its anti-inflammatory properties [79,80]. Neuroglobin expression has been reported in similar regions for both sexes, while the co-localization rate of the neuroglobin with Iba1 immunoreactive cells is doubled in males compared to females [61].

Other studies have focused on demonstrating that clusters of differentiation (CD) 4-positive cells in females are intrinsically oriented to proliferate and produce higher levels of Interferon gamma (IFNγ) and lower levels of IL-17A than male T-cells. This sexual difference is probably related to androgens (testosterone in particular) that are present in different levels in the two *genera* [80,81]. The immune response can be altered in many ways by sex hormones; however, it is still poorly understood how endogenous hormone levels affect its quality and quantity. The gene expression of cytokines seems to be affected by hormones; however, the mechanism is unclear. Intriguingly, the IFNγ gene seems to be directly influenced by 17-beta-estradiol. In fact, the hormone greatly enhances the activity of the IFNγ promoter in the lymphoid cells expressing the appropriate hormone receptor, resulting in an increase in the effect of T cell-activating agents [82].

## 6. Conclusions

It is known that microglial cells are critically involved in mediating pain behavior in different preclinical models of neuropathic pain. However, the evidence of implication of these cells in pain remains confined to male rodents. Even if microglia assume an activated phenotype following nerve damage in both sexes, they may not be crucially involved in facilitating neuropathic pain behavior in females. Recent findings suggest that peripheral immune cells play a role in pain processing, which appears to be associated with T-cell recruitment. Altogether, the evidence supports the hypothesis of a sex-dependent establishment of neuroinflammation-driven allodynia, opening up a new scenario that was previously neglected. Further studies are needed to clarify the mechanisms through which these processes occur in order to employ more effective therapeutic strategies for treating such pathologies.
